# Baicalin suppresses lung cancer growth phenotypes via miR-340-5p/NET1 axis

**DOI:** 10.1080/21655979.2021.1922052

**Published:** 2021-05-06

**Authors:** Fucheng Zhao, Zhenxia Zhao, Yanru Han, Sujuan Li, Caili Liu, Kui Jia

**Affiliations:** Department of Integrated Chinese and Western Medicine, The First Affiliated Hospital of Xinxiang Medical University, Weihui, Henan, China

**Keywords:** Lung cancer, baicalin, miR-340-5p, NET1

## Abstract

As a malignant disease, lung cancer has a high morbidity and mortality rate. Baicalin is derived from Radix Scutellariae and has anti-tumor effects, however, its role in lung cancer remains unknown. Here, functional assays suggested baicalin suppressed in vitro lung cancer phenotypes. We used micro (mi)RNA array analysis to explore baicalin effects on miRNA expression. We observed baicalin increased miR-340-5p expression, whereas inhibition of this expression abolished anti-tumor effects of baicalin. Furthermore, neuroepithelial cell transforming 1 (NET1) functioned as a miR-340-5p target, and acted in a baicalin-dependent manner to regulate lung cancer progression. Thus, baicalin elicited antitumor activities by affecting the miR-340-5p/NET1 axis, suggesting a new approach to lung cancer clinical management.

## Introduction

Globally, lung cancer is an extremely common malignancy, with an increased death rate [1]. Non-small cell lung cancer accounts for an 85–90% prevalence in lung cancer patients [2,3]. While therapeutic approaches have increased in recent decades, the disease recurrence rate is high, and the 5-year overall-survival rates is still low [4,5]. Thus, it is urgent to determine the underlying mechanism and explore an effective treatment to overcome this malignancy.

In recent years, traditional Chinese medicines (TCMs) as lung cancer therapies have garnered considerable research attention. Increasingly, studies have indicated that TCMs efficiently limit postoperative cancer complications, accelerate postoperative recovery, and reduce metastasis recurrence [6–8]. Recently, Gao et al found that Sinomenine limited breast cancer phenotypes via miR-29/PDCD-4 modulation [9]. Yu et al found that Rosmarinic acid decreased gastric cell cancer resistance to 5-fluorouracil by affecting miR-6785-5p/FOXO4 [10]. Cui et al showed that Astragaloside IV reduced liver cancer phenotypes by affecting miR-150-5p/β-catenin [11]. However, the therapeutic effects and underlying mechanism of TMCs in tumor progression remain largely unclear.

Baicalin (β-d-glucopyranosiduronic acid, 5, 6-dihydroxy-4-oxo-2-phenyl-4 H-1-benzopyran-7-yl), which is a lipophilic flavonoid glycoside isolated from Radix Scutellariae [12], with antioxidant, anti-inflammatory, neuro-protective, antivirus, and anti-tumor effects [13,14]. Yang et al found that Baicalin alleviated interleukin (IL)-1 β-induced inflammation by down-regulating miR-126 in chondrocytes [15]. Wang et al found that Baicalin induced colon cancer cell senescence by up-regulating DEPP and activating the Ras/Raf/MEK/ERK pathway [16]. Yu et al found that baicalin reduced prostate cancer growth and arrested cell-cycle functions by regulating CDK6/FOXM1 [17]. However, baicalin mediated anti-lung cancer effects and underlying mechanisms remain unclear.

In this research, the antitumor activity and underlying mechanism of baicalin on lung cancer cells was investigated. Our results showed that the anti-tumor effects of Baicalin on lung cancer growth characteristics were through modulation the miR-340-5p/neuroepithelial transforming gene 1 (NET1) partnership. Our research promotes baicalin as an effective anti-tumor molecule in lung cancer development.

## Materials and methods

### Cell growth and transfection methods

Lung cancer cell lines (A549, H1299) were provided by the American Type Culture Collection (ATCC, Manassas, VA, USA) and maintained in RPMI-1640 supplemented with 10% fetal bovine serum (FBS; HyClone, Logan, UT, USA), 100 U/ml penicillin, and 100 μg/ml streptomycin at 37°C in a 5% CO_2_ incubator.

MiR-340-5p mimics, miR-340-5p inhibitors, pcDNA3.1-NET1 and their respective controls were compounded by GenePharma (Shanghai, China). Lipofectamine 2000 (Thermo Fisher Scientific, Waltham, MA, USA) was used for transfections in line with the protocols.

### Cell viability

The cell viability was measured using the cell counting kit-8 (CCK-8, Dojindo, Kumamoto, Japan). Cells in 96-well plates were supplemented with CCK-8 solution for 120 min at 37°C. A microplate reader (Bio-Rad, Hercules, CA, USA) measured absorbance at 450 nm.

### Colony formation assay

Treated cells (1000 cells/well) were plated in 6-well plates and cultured in growth medium for 2 weeks. Colonies were processed by fixing in paraformaldehyde, staining in 0.1% crystal violet, and enumerated by inverted microscopy (Olympus, Tokyo, Japan).

### Flow cytometry analysis

FITC Annexin V Apoptosis Detection kit (Beyotime, Shanghai, China) and cell-cycle assay kit (Vazyme, Nanjing, China) were used for apoptosis and cell cycle analyses according to manufacturer’s instructions, and previous work [16]. The apoptotic rate and cell cycle were analyzed by flow cytometry (BD Biosciences, San Jose, CA, USA)

### Cell invasion assay

Cell invasion was measured by Transwell chambers coated with 50 µl of Matrigel (8-µm pores; BD Biosciences, San Jose, CA, USA). Cells were grown in upper chambers containing 200 µl serum free medium, with 750 µl 10% complete medium in lower chambers. After 2 days, upper membrane cells were removed, and invaded cells processed and enumerated by inverted microscopy (magnification, × 200; Olympus, Japan).

### Quantitative real-time polymerase chain reaction (qRT-PCR)

RNA was extracted from cells using TRIzol reagent (Invitrogen, Carlsbad, CA, USA) and reverse transcribed with a reverse transcription kit TaKaRa, Dalian, China). Quantitative RT-PCR, using a SYBR green kit (Applied Biosystems, Foster City, CA, USA) was performed on ABI 7900HT equipment. Gene expression was calculated using the 2^–ΔΔCT^ method, GAPDH and U6 were used as internal references, respectively.

### Dual-luciferase reporter assay

NET1 wild-type (NET1-Wt) and NET1 mutant-type (NET1-MUT) were ligated into a pmir-GLO dual-luciferase vector (Promega, Madison, WI, Wisconsin). The constructed luciferase reporter plasmids were co-transfected with miR-340-5p mimics or miR-NC into HEK293T cells planted in a 96-well plate. After two days, luciferase activity was assessed by using the Dual Luciferase Reporter Assay System (Promega, Madison, WI, USA). The relative luciferase activity was normalized to Renilla luciferase activity.

### Western blot

Proteins were processed using RIPA buffer, and quantified by BCA assay (Tiangen, Shanghai, China). Approximately equal amounts of protein samples were separated by 10% SDS-PAGE and transferred to PVDF membranes (Millipore, Bedford, MA, USA). Membranes were blocked in 3% BSA and then overnight incubation with primary antibodies (anti-NET1, ab113202) (Abcam, Cambridge, UK) at 4°C. After washing in TBST (3 ×), HRP-conjugated secondary antibodies were added, after which enhanced chemiluminescence (ECL) reagent (Millipore, Billerica, MA, USA) captured protein signals. GAPDH (anti-GAPDH, ab37168) was used to normalize signals.

### Statistics

Data were represented as mean ± standard deviation (SD). All experiments in this study were repeated at least 3 times independently. Differences were calculated by Student’s t test or one way ANOVA, followed by Tukey’s test. A P < 0.05 value indicated significant differences. SPSS 18.0 software (SPSS, Inc., Chicago, IL, USA) was used for data processing.

## Results

### Baicalin reduces A549 and H1299 cells proliferation and invasion

**Figure 1.**
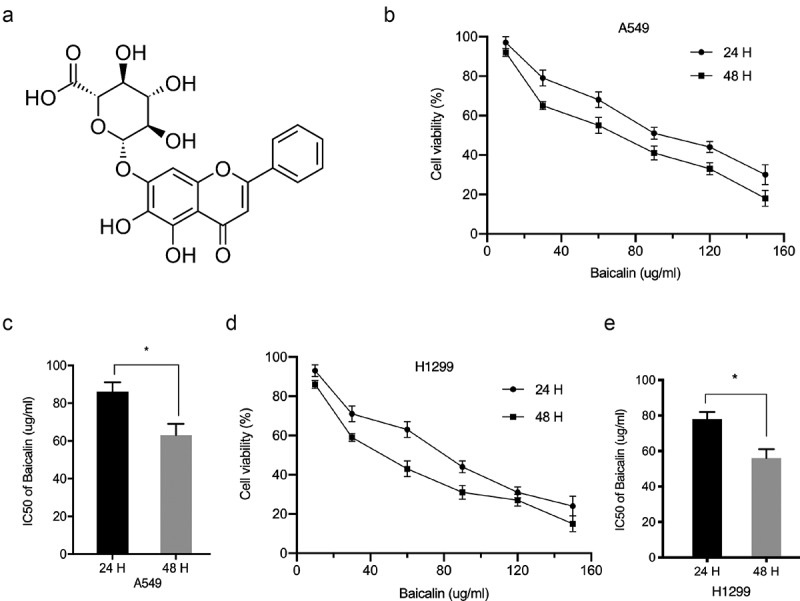
Baicalin reduced A549 and H1299 cell viability. (a) The baicalin chemical structure. (b-e) Cell viability was analyzed using different baicalin concentrations (2 time-points). *P < 0.05

**Figure 2.**
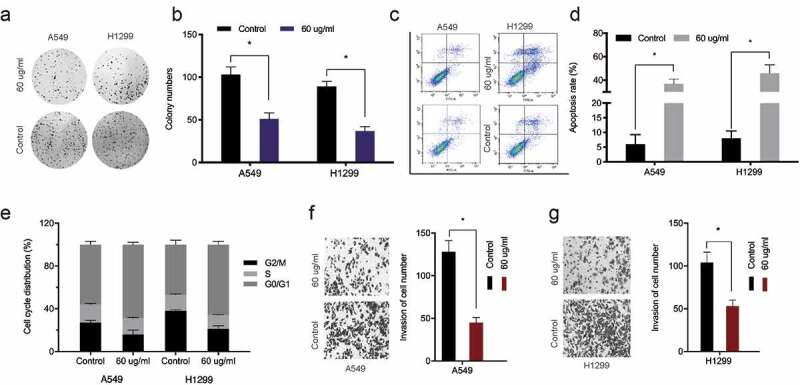
Baicalin reduced A549 and H1299 cell phenotypes. (a, b) Baicalin effects on cell growth by colony formation assay. (c-e) Baicalin effects on apoptosis and cell cycle by flow cytometry. (f-g) Baicalin effects on invasion by transwell assay. *P < 0.05

The chemical structure of baicalin is shown in Figure 1a. We investigated baicalin effects on lung cancer cells proliferation and invasion. After treatment with 10, 30, 60, 90, 120, and 150 µg/ml baicalin concentrations [18] at two time points (24 h and 48 h), cell proliferation was reduced, and efficacy was dose- and time-dependent (Figure 1b-e). Colony formation data indicated baicalin significantly limited in vitro cell proliferation (Figure 2a and 2b). Next, the impact of baicalin on apoptosis and the cell cycle was assessed; apoptosis was significantly upregulated and the cell-cycle arrested in the G1/S phase in baicalin treated cells (Figure 2c-e). Additionally, transwell assays indicated baicalin significantly reduced in vitro cell invasion (Figure 2f and 2g). These data indicated that baicalin restrained the proliferation and invasion process of lung cancer cells.

### MiR-340-5p impacts on the anti-tumor effects of baicalin

**Figure 3.**
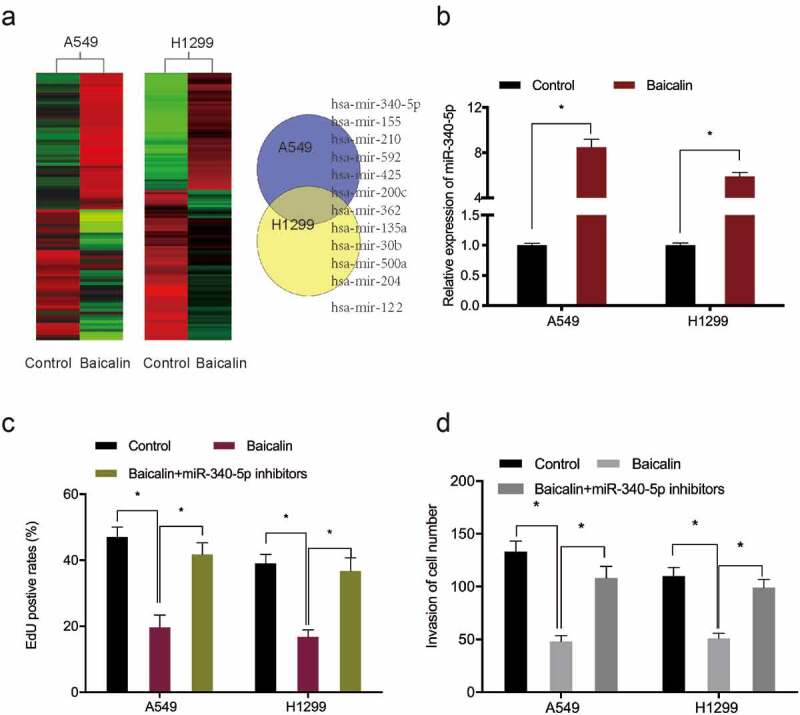
MiRNA expression profiles using miRNA microarray. (a) Clustering of differentially expressed miRNAs. (b) Baicalin raised miR-340-5p levels in A549 and H1299 cells. (c, d) MiR-340-5p inhibition recovered baicalin effects toward key cell phenotypes (proliferation and invasion). *P < 0.05

To investigate baicalin mediated mechanisms, miRNA profiles in baicalin treated cells were microarray tested, with miR-340-5p significantly up-regulated in these cells (Figure 3a). Quantitative RT-PCR results further confirmed miR-340-5p significantly up-regulated in baicalin treated lung cancer cells (Figure 3b). EdU data revealed that miR-340-5p inhibition recovered baicalin effects toward lung cancer cells proliferation (Figure 3c). Moreover, this inhibition abrogated baicalin effects on lung cancer cells invasion characteristics (Figure 3d). Combined, these results suggested that baicalin treatment might reduce growth characteristics by upregulating miR-340-5p expression.

### MiR-340-5p directly targets NET1

**Figure 4.**
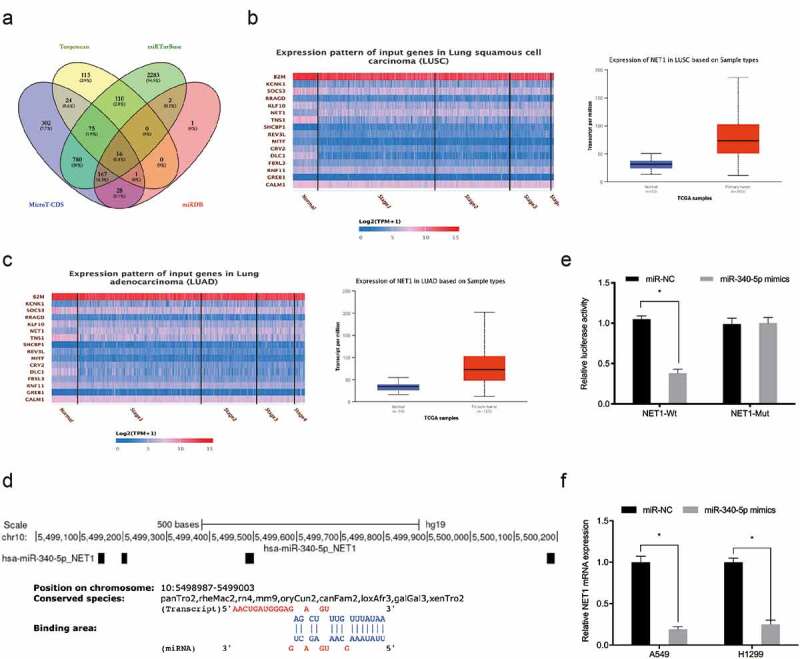
MiR-340-5p targeted NET1. (a) Venn diagrams showing potential miR-340-5p targets. (b, c) Potential target genes expression in the TCGA database. (d) NET1 binding sites in miR-340-5p. (e) Dual-luciferase assays verified the targeting relationship between miR-340-5p and NET1. (f) Overexpressed miR-340-5p reduced NET1 mRNA levels. *P < 0.05

To further investigate the underlying mechanism of miR-340-5p in lung cancer, Targetscan, MicroT-CDS, miRTarBase, and miRDB [19–22] were used to investigate miR-340-5p molecular targets (Figure 4a). These potential target genes expression was then explored in the TCGA database, and NET1 was chosen for further analysis (Figure 4b-d). Luciferase assays suggested that miR‐340-5p mimics observably attenuated the luciferase activity of NET1‐Wt group but not the mutant group (Figure 4e). Quantitative RT-PCR showed miR-340-5p mimics significantly decreased NET1 expression in lung cancer cells (Figure 4f). Thus, these results demonstrated that NET1 was the target of miR-340-5p.

### NET1 up-regulation reverses baicalin effects

**Figure 5.**
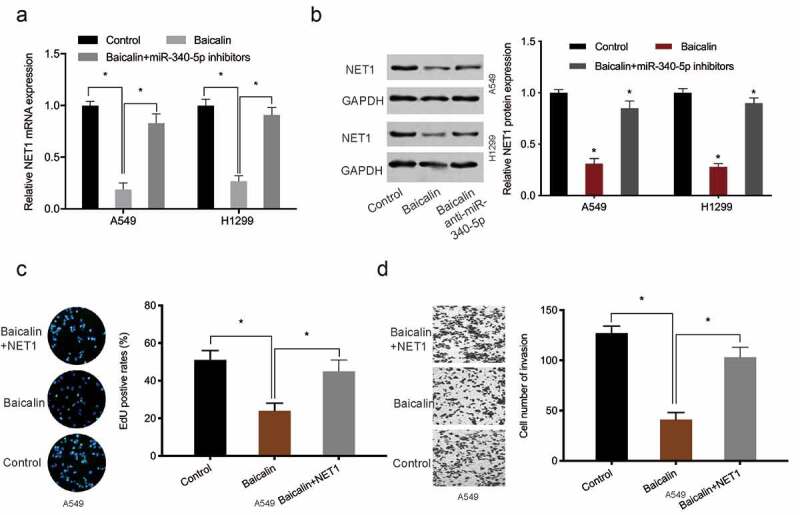
NET1 restoration reversed the effects of baicalin. (a, b) MiR-340-5p inhibition reversed the effects of baicalin on NET1 levels in lung cancer cells. (c, d) NET1 overexpression reversed the suppression effects of baicalin on lung cancer cells phenotypes (proliferation and invasion). *P < 0.05

**Figure 6.**
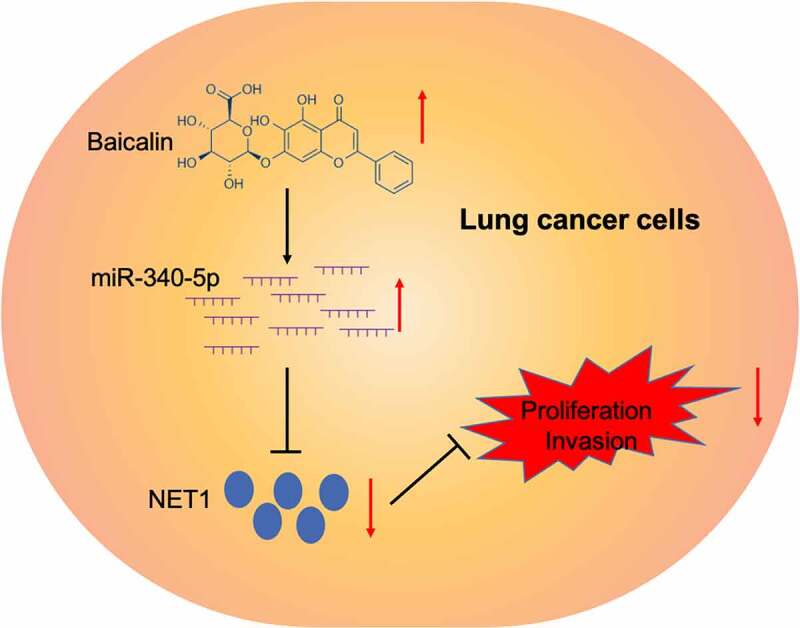
Graphic abstract: Baicalin suppressed lung cancer growth phenotypes via miR-340-5p/NET1 axis

To investigate whether baicalin functioned via the miR-340-5p/NET1 axis, qRT-PCR and Western blot data indicated baicalin decreased NET1 expression, whereas these affects could be abrogated by miR-340-5p inhibition (Figure 5a and 5b). Next, functional rescue assays showed that overexpression NET1 reversed the suppressive effects of Baicalin on lung cancer cells proliferative, and invasive capabilities (Figure 5c-d). Combined, baicalin reduced these phenotypes by targeting the miR-340-5p/NET1 axis (Figure 6).

## Discussion

Baicalin, a lipophilic flavonoid glycoside isolated from Radix Scutellariae, has great potential to be used for various cancers, such as pancreatic cancer, ovarian cancer, and prostate cancer et al [23–25]. Recently, increasing studies revealed that baicalin exerted key effects during lung cancer development. For example, Diao et al showed that baicalin reduced lung cancer growth by regulating the PBK/TOPK axis [26]. You et al found that baicalin suppressed in vitro lung cancer growth and metastasis by activating the SIRT1/AMPK axis [27]. In the present study, we also found that baicalin restrained the proliferation and invasion process of lung cancer cells in vitro. However, the underlying mechanisms of baicalin in lung cancer progression remain unclear.

MicroRNAs (miRNAs) are a class of short endogenous non-coding RNAs, which act as a post-transcriptional regulator via binding to the 3ʹuntranslated region of its target genes [28]. Increasingly, they are exhibiting vital functions in several diseases, including cancer [29]. Here, miRNA microarray showed that miR-340-5p was significantly upregulated in the Baicalin-treated lung cancer cells, and the data was further confirmed by qRT-PCR. Furthermore, miR-340-5p inhibition attenuated these baicalin anti-tumor effects in lung cancer cells. Thus, we demonstrated that miR-340-5p overexpression may exert critical roles in baicalin anti-tumor effects in lung cancer progression.

The neuroepithelial cell transforming gene 1 (NET1), a RhoA guanine nucleotide exchange factor, was firstly isolated from neuroepithelioma cells [30]. Recent studies showed that NET1 play key roles in tumor development. For example, Sun et al showed that NET1 promoted acute lymphoblastic leukemia cell proliferation and chemoresistance [31]. Xiao et al found that miR-22-3p enhanced multi-chemoresistance by targeting NET1 in bladder cancer cells [32]. Zong et al revealed that lncRNA CTC-497E21.4 promoted gastric cancer progression via the miR-22/NET1 regulation [33]. Nevertheless, the roles and underlying mechanisms of NET1 in lung cancer are still unexplored. Here, NET1 was identified as a miR-340-5p target gene. Luciferase activity indicated NET1-Wt was reduced by miR-340-5p mimics, whereas miR-340-5p overexpression dramatically reduced NET1 expression in lung cancer cells. Importantly, miR-340-5p inhibition eliminated suppressive baicalin effects toward NET1 expression. Rescue assays revealed NET1 overexpression reversed the effects induced by baicalin on lung cancer cells phenotypes.

## Conclusion

In summary, baicalin suppressed lung cancer progression by affecting the miR-340-5p/NET1 axis, suggesting a potential therapeutic target for the treatment of lung cancer.

## Supplementary Material

Supplemental Material

## Data Availability

The dataset supporting the conclusions of this article is included within the article.

## Highlights

1. Baicalin reduces lung cancer cells proliferation and invasion
2. MiR-340-5p directly targets NET1
3. Baicalin reduces lung cancer cells phenotypes by targeting the miR-340-5p/NET1 axis
