# Supplementary figures and images for: Baicalin suppresses lung cancer growth phenotypes via miR-340-5p/NET1 axis
Source: Bioengineered. 2021 May 6;12(1):1699–707. doi: 10.1080/21655979.2021.1922052 (PMC8806212; doi:10.1080/21655979.2021.1922052)

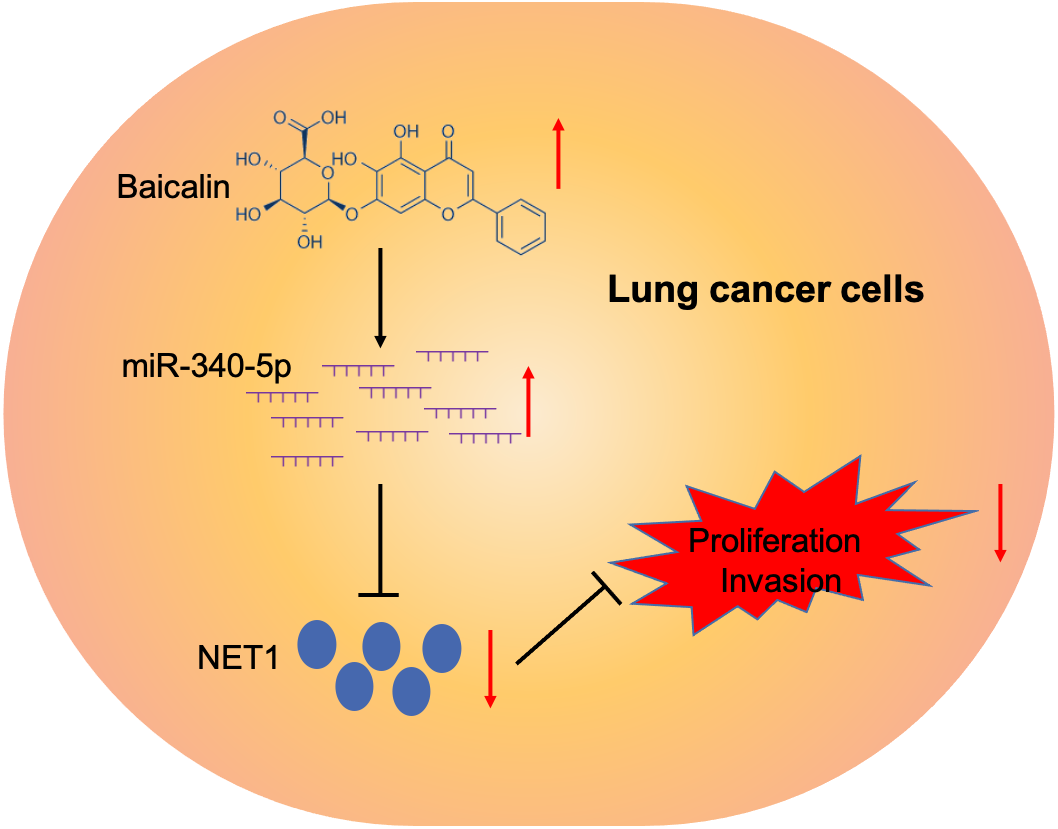

Supplement: Supplemental Material [file KBIE_A_1922052_SM5347.png]
